# Determinants of knowledge and prevention measures towards COVID-19 pandemic among Lebanese dentists: a cross sectional survey

**DOI:** 10.1186/s12903-021-01599-9

**Published:** 2021-05-06

**Authors:** Mira Hleyhel, Christine Haddad, Nour Haidar, Maria Charbachy, Nadine Saleh

**Affiliations:** 1 CERIPH, Center for Research in Public Health, Pharmacoepidemiology Surveillance Unit, Faculty of Public Health II, Lebanese University, Fanar, Lebanon; 2 INSPECT-LB: Institut National de Santé Publique, Epidémiologie Clinique Et Toxicologie - Liban, Beirut, Lebanon; 3 Faculty of Public Health, Lebanese University, Fanar, Lebanon

**Keywords:** SARS-CoV-2, Health knowledge, Prevention, Dental practice, Pandemic

## Abstract

**Background:**

Coronavirus Disease 2019 (COVID-19) is a major global threat. Healthcare professionals including dentists are facing real challenges during this pandemic. This study aimed to evaluate knowledge, attitudes, and prevention measures of Lebanese dentists towards COVID-19 and determinants of high level of knowledge and prevention practices.

**Methods:**

A cross-sectional study was conducted between May and August 2020 in Lebanon on a random sample of 323 Lebanese dentists. Data were collected through an online survey questionnaire. A multivariate linear regression model was used to evaluate factors associated with COVID-19 knowledge. A multivariate logistic regression was conducted to evaluate the factors associated with high level of prevention measures towards COVID-19.

**Results:**

The mean COVID-19 knowledge index was 24.5 over 38 with only 15% achieving high knowledge level. The mean prevention measures index was 11.4 over 16 with only 35% achieving high prevention level. Higher knowledge index was associated with younger age, being employed, and considering dentist’s role significant in teaching others about COVID-19. General dental practitioners, dentists living with family members and concerned about their family members to get infected because of their occupational exposure were more likely to report higher level of adopted prevention measures. Higher knowledge was associated with high level of prevention measures.

**Conclusions:**

Given the rapid evolution of information related to COVID-19 pandemic, dentists should be regularly educated through trainings, workshops, and updates of national guidelines for dental healthcare.

## Introduction

The World Health Organization has declared Coronavirus Disease 2019 (COVID-19) a pandemic on March 11, 2020, considering it a worldwide public health emergency [1, 2]. COVID-19 is caused by a novel coronavirus, called severe acute respiratory syndrome coronavirus 2 (SARS-CoV-2). As of February 2021, the worldwide global cumulative number of cases was estimated at 105.4 million  with 2.3 million cases of deaths [3]. In Lebanon, as of February 15, 2021, and since the start of the pandemic, an estimated 340,861 SARS-CoV-2 cases and 4037 deaths were recorded with a fatality rate of 1.2% [4].

Until now, there is still no universal effective treatment acting specifically against COVID-19 [5]. For this, adopting the appropriate preventive measures remains one of the best solutions to fight against the infection. Since the emergence of SARS-CoV-2, healthcare professionals have been facing critical challenges. Dental professionals who are in close contact with the patient, are at great risk of exposure to the virus, while performing procedures and surgical acts that produce a mixture of splatter, droplets, and aerosols. Thus, it is important to evaluate dentists’ knowledge and prevention measures towards COVID-19 as it is highly likely that the current pandemic will impact dental practice in general and will shape the training of the future generations of dentists. A contribution to the fight against the pandemic is expected from dentists through spreading awareness, screening for suspected cases, providing consultations and through updating dentistry curricula to include trainings about new methods of prevention control and about preparedness for future pandemics [6].

To control the spread of the virus in dental healthcare facilities, the Centers for Disease Control and Prevention [7, 8], and the American Dental Association [9] have published different guidelines for dental healthcare practice. In Lebanon, dental healthcare guidelines [10] had been released by the Lebanese Dental Association (LDA) at the time when data started to be collected for the study. Those guidelines highlighted the types of personal protective equipment (PPE) required and their appropriate use, the definition of a dental emergency, and included a protocol for dental care during COVID-19 pandemic.

COVID-19, which is a highly contagious disease, constitutes a major threat to the health of dentists, their assistants, and patients. Therefore, dentists should have information and should be aware of the most effective prevention measures to protect their owns and patients’ health. Given their crucial role in combatting the outbreak of the disease, we aimed in this study to assess the knowledge, attitudes, prevention measures and clinical practices of Lebanese dentists regarding COVID-19 pandemic.

## Methods

### Study design and sampling

A cross-sectional survey was conducted from May to August 2020 using a web-based questionnaire. A stratified random sampling from the list of all Lebanese dentists registered in the LDA was conducted. For this study, 10% of dentists’ population was taken as sample size estimated at 320 dentists. The sample was proportionally distributed according to the Lebanese governorates (Beirut, Mount Lebanon, North Lebanon, South Lebanon and Bekaa). Assuming that the rate of completed questionnaires will be 20% [11], the total number of dentists to be selected and invited to participate were 1600 dentists.

### Questionnaire and data collection

The questionnaire was designed in English and then translated to French and reviewed for consistency. The questions were developed based on the literature and international guidelines. The questionnaire included:

1. Sociodemographic characteristics and factors related to participants general practices and health.
2. Knowledge assessed by 7 questions including 38 answers on: clinical symptoms, incubation period, modes of transmission, molecules under investigation in clinical trials and mouthwash virucidal activity against the COVID-19 virus.
3. Prevention measures adopted in dental clinics and other practices.
4. Attitudes towards COVID-19 pandemic

Each participant was contacted by phone call where the survey objective was described. After obtaining an oral informed consent from the participant, a google form link was sent through phone message or whatsapp.

### Statistical analysis

Descriptive analysis was used to describe participant characteristics using means and standard deviations (SD) for quantitative variables and frequencies and percentages for qualitative variables. For knowledge index, a correct answer was given one point and wrong or “I don’t know” was given zero point. The total knowledge index was calculated by summing all the answers and ranged between 0 and 38. The index was classified into 3 levels of knowledge, high (80–100%), moderate (60–79%) or low (≤ 59%) level [12]. The same method was used to calculate and categorize the prevention measures index which ranged between 0 and 16.

A multivariate linear regression model was performed to identify factors associated with the knowledge index. A multivariate logistic regression model was used to explore factors associated with high prevention measures level taking low and moderate index combined as the reference category. Independent variables associated with the dependent variables with a p-value ≤ 0.2 in the bivariate analyses were included in the multivariate models [13]. Data was analyzed using SPSS software.

## Results

### Characteristics of the study participants

A total of 323 dentists completed the questionnaire. The age of the participants ranged between 25 and 70 years old, with more than half (57.3%) being males. Around a quarter of the participants were general dental practitioners, most of them had more than 10 years of experience, and only 17.6% had comorbidities. A detailed description of study participants characteristics is presented in Table 1.

**Table 1:** Characteristics of study participants (n = 323)

Characteristics	Mean ± SD	N (%)
**Age (years)**	44.62 ± 10.8	
**Gender**		
Male		185 (57.3%)
Female		138 (42.7%)
**Work region**		
Beirut		64 (19.8%)
Mount Lebanon		178 (55.1%)
Bekaa, South and North of Lebanon		81 (25.1%)
**Graduation university**		
Public: LU		117 (36.2%)
Private: USJ or BAU		112 (34.7%)
Abroad		94 (29.1%)
Years of experience*		
≤ 10		88 (27.2%)
> 10		231 (71.5%)
**Specialty (1)**^†^		
General practitioner		155 (48%)
Oral surgeon		64 (19.8%)
Implant surgeon		43(13.3%)
Orthodontist		30(9.3%)
Endodontic		29 (9%)
Periodontic		25 (7.7%)
Prosthodontic		22 (6.8%)
Restorative Dentist		18(5.6%)
Other		21 (6.5%)
**Specialty (2)**		
General practitioner only		84(26%)
Surgeon (oral, implant)		104(32.2%)
Specialist dentist		135(41.8%)
**Employment status**		
Self-employed		221(68.4%)
Employee		95 (29.4%)
Unemployed		7 (2.2%)
**Living with family members**		
Yes		233 (72.1%)
No		90(27.9%)
**Comorbidities**^†^		
None		268 (83%)
Hypertension		20 (6.2%)
Diabetes		18 (5.6%)
Cardiovascular disease		9 (2.8%)
Obesity		10 (3.1%)
Respiratory disease		5 (1.5%)
Active cancer		2 (0.6%)
Immunodeficiency		5 (1.5%)

^*^Years of experience: missing data = 4

^†^The sum of the percentages is greater than 100% because dentists can have several specialties and several comorbidities

*LU* Lebanese University, *USJ* Saint-Joseph University, *BAU* Beirut Arab University

### Knowledge towards COVID-19

Almost all the respondents reported the main COVID-19 symptoms which included fever (99.1%), fatigue (91.3%), dry cough (92.6%) and headache (92.9%). However less than half reported the less common symptoms. Most of the dentists (73%) reported the correct average incubation period of the virus being 1–14 days and identified the modes of transmission of the virus. Regarding molecules under investigation in clinical trials, 90.7% of the respondents reported hydroxychloroquine, meanwhile, less than half of the respondents reported other molecules (Table 2). The mean knowledge index regarding COVID-19 was 64.5% and its Cronbach alpha for its internal consistency was 0.79. Low and moderate knowledge was observed in around 85% of the participants (Table 3).

**Table 2:** Responses to questions about Coronavirus Disease 2019 (COVID-19) knowledge by the participating dentists

	Answers	N (%)
**Incubation period (days)**		
1–14	Correct	238 (73.7)
2–7	Incorrect	28 (8.7)
7–14	Incorrect	25 (7.7)
7–21	Incorrect	25 (7.7)
I don’t know	Incorrect	7 (2.2)
**Symptoms**		
Fever	Correct	320 (99.1)
Fatigue	Correct	295 (91.3)
Dry cough	Correct	299 (92.6)
Headache	Correct	300 (92.6)
Shortness of breath	Correct	296 (91.6)
Diarrhea	Correct	155 (48)
Vomiting	Correct	134 (41.5)
Runny nose	Correct	161 (49.8)
Sore throat	Correct	240 (74.3)
Red eyes	Correct	98 (30.3)
Skin rash	Correct	137 (42.4)
Joint or muscle pain	Correct	197 (61)
Loss of smell and taste	Correct	263 (81.4)
May present with no symptoms	Correct	308 (95.4)
I don’t know	Incorrect	1 (0.3)
**Mode of transmission**		
Droplets	Correct	310 (96)
Coughing and sneezing	Correct	319 (98.8)
Hand shaking	Correct	264 (81.7)
Touching surfaces such as doorknobs and tables	Correct	257 (79.6)
Contact with blood	Incorrect	169 (52.3)
Contact with stools	Incorrect	71 (22)
I don’t know	Incorrect	1 (0.3)
**Transmission is possible when**		
Symptomatic	Correct	300 (92.9)
Asymptomatic	Correct	217 (67.2)
Asymptomatic with positive PCR	Correct	300 (92.9)
I don’t know	Incorrect	7 (2.2)
**Molecules under investigation in clinical trials**		
Chloroquine	Correct	156 (48.3)
Hydroxychloroquine	Correct	293 (90.7)
Azithromycin	Correct	100 (31)
Ivermectin	Correct	49 (15.2)
Remdesivir	Correct	124 (38.4)
Lopinavir	Correct	92 (28.5)
Ritonavir	Correct	89 (27.6)
Fluoroquinolones	Incorrect	12 (3.7)
Methylprednisolone	Correct	9 (2.8)
Penicillin	Incorrect	10 (3.1)
Oseltamivir	Incorrect	11 (3.4)
Thalidomide	Correct	6 (1.9)
I don’t know	Incorrect	14 (4.3)
**COVID-19 symptoms often resolve with time and do not require any special treatment**		
True	Correct	250 (77.4)
False	Incorrect	58 (18)
I don’t know	Incorrect	15 (4.6)
**All mouthwash has a high virucidal activity against COVID-19**		
True	Incorrect	99 (30.7)
False	Incorrect	55 (17)
There is a lack of studies	Correct	139 (43)
I don’t know	Incorrect	30 (9.3)

**Table 3:** Knowledge and prevention measures indexes of the participants regarding Coronavirus Disease 2019 (COVID-19)

Characteristic	Value
**Knowledge**	
**Range of index achieved**	12–34
**Mean ± SD**	24.51 ± 4.65
**Level of knowledge**	
Low (≤ 59%) n (%)	104 (32.2)
Moderate (60–79%) n (%)	170 (52.6)
High (80–100%) n (%)	49 (15.2)
**Prevention measures**	
**Range of index achieved**	4–16
**Mean ± SD**	11.39 ± 2.61
**Level of prevention measures**	
Low (≤ 59%) n (%)	68 (21.1)
Moderate (60–79%) n (%)	143 (44.3)
High (80–100%) n (%)	112 (34.7)

### Prevention measures towards COVID-19

Almost all the respondents reported adopting the right preventive measures in the dental clinics by applying hand hygiene (95.7%), disinfection for surfaces (90.4%) use of PPE (93.2%) and protective clothes for staff members (84.8%). More than half of the respondents reported that they use high-speed evacuation for dental procedures (65%), avoid operations that can produce droplets or aerosols (57.6%) and avoid moving patients out of their area unless necessary (54.8%). Only 30.7% of respondents reported placing patients with known or suspected COVID-19 infection in adequately ventilated single rooms (Table 4a).

**Table 4:** Prevention Measures adopted by the participating dentists in the dental clinic to prevent the transmission of Coronavirus Disease 2019 (COVID-19)

	N (%)
**a. Prevention measures adopted in the dental clinic**	
Use high-speed evacuation (saliva evacuator) for dental procedures producing an droplets or aerosols	210 (65%)
Avoid operations that can produce droplets or aerosols	186 (57.6%)
Frequently clean hands by using alcohol-based hand rub or soap and water	309 (95.7%)
Routinely clean and disinfect surfaces in contact with known or suspected patients	292 (90.4%)
Use personal protective equipment (such as dental goggles, masks, and gloves) only with patients with known or suspected COVID-19 infection	301 (93.2%)
Avoid moving and transporting patients out of their area unless necessary	177 (54.8%)
All health staff members wear protective clothing	274 (84.8%)
Place patients with known or suspected patients COVID-19 infection in adequately ventilated single rooms	99 (30.7%)
**b. Measures adopted with patients in the dental clinic**	
Ask patients to sit far from each other	249 (77.1%)
Ask patients to wear masks while in the waiting room	314 (97.2%)
Ask patients to wash hands before getting in the dental chair	285 (88.2%)
Take patient’s temperature when they arrive at the clinic	294 (91%)
Screen patients for signs and symptoms of COVID-19 infection	229 (70.9%)
Ask patients about recent travel	203 (62.8%)
Ask patients about recent contact with a person with known or suspected COVID-19 infection	138 (42.7%)
Use a rubber dam	122 (37.8%)

Most dentists reported adopting the right prevention measures with the patients, by asking them to sit far from each others (77.1%), wearing masks (97.2%), applying hand hygiene (88.2%), screening patients (70.9%), taking their temperature (91%) and asking about recent travel (62.8%). Meanwhile, less than half of the respondents reported asking patients about recent contact with suspected or known infected people (42.7%) and the use of rubber dam for dental procedures (37.8%) (Table 4b).

Overall, the mean prevention measures index of the participants was 71.2% and the Cronbach alpha for its internal consistency was 0.67. While 21.1% of dentists reported adopting low prevention measures level, 34.7% reported adopting high level of prevention measures towards COVID-19 (Table 3).

### Other practices and attitudes towards COVID-19

Around half of the respondents reported treating only dental emergency cases during lockdown (55.7%), accepting to treat patients with flu like symptoms (48.6%) and avoiding treating patients with known or suspected COVID-19 infection (51.7%). Dentists reported opening their clinics on average of 1.1 days (SD = 1.3) per week during lockdown. Almost all the respondents reported the prescription of paracetamol, 38% reported the prescription of nonsteroidal anti-inflammatory drugs and only 14% reported the prescription of corticosteroids for pain management during COVID-19 pandemic.

While 18.6% of the respondents reported perceiving COVID-19 as very dangerous, 46.4% and 35% reported perceiving it as moderately dangerous and not dangerous respectively. Approximately half of the respondents reported that their role in teaching others about COVID-19 is very significant and only 12.4% reported that their role is not significant at all. Lastly, most dentists reported being afraid of getting infected with COVID-19 (66.6%) and being afraid about family members to get infected due to their occupational exposure (79.3%).

### Factors associated with knowledge towards COVID-19

The multivariate linear regression model showed that higher knowledge index was associated with working in Mount Lebanon and considering dentist’s role important in teaching others about COVID-19. Older age and being self-employed compared to being employed were associated with a lower knowledge index (Table 5a).

**Table 5:** Factors associated with knowledge and prevention measures indexes toward Coronavirus Disease 2019 (COVID-19) among participating dentists

	Unstandardized Beta	95% CI of unstandardized Beta	p-value
**a. Multivariate linear regression for the factors associated with knowledge index**			
**Age (years)**	-0.15	-0.20; -0.09	** < 0.001**
**Work region**			
Beirut	ref		
Mount Lebanon	2.66	1.54; 3.78	** < 0.001**
Others	-0.44	-1.35; 1.26	0.947
**Employment status**			
Employee	ref		
Self-employed	-1.12	-2.16; -0.09	**0.034**
Un-employed	2.74	-0.16; 5.64	0.064
**Dentist’s role in teaching others about COVID-19**			
Not significant	ref		
Moderately significant	2.88	1.39; 4.36	** < 0.001**
Very significant	2.38	0.92; 3.84	**0.001**

	Adjusted OR	95% CI of the adjusted OR	p-value
**b. Multivariate logistic regression for the factors associated with high prevention measures index (low and moderate combined as a reference)**			
**Work region**			
Beirut	ref		
Others	0.74	0.35; 1.56	0.427
Mount Lebanon	0.34	0.17; 0.69	**0.003**
**Specialty**			
General practitioner	ref		
Surgeon	0.59	0.31; 1.15	0.121
Specialist	0.48	0.25; 0.91	**0.024**
**Living with family members**			
No	ref		
Yes	2.31	1.23; 4.34	**0.010**
**Dentist’s role in teaching others about COVID-19**			
Not significant	ref		
Moderately Significant	2.76	1.04; 7.31	**0.041**
Very Significant	2.13	0.82; 5.58	0.123
**Afraid about family members to get infected with COVID-19**			
No	ref		
Yes	2.05	1.00; 4.19	**0.050**
**Knowledge index**	1.15	1.08; 1.23	** < 0.001**

*Beta* regression coefficient**,** *CI* confidence interval, *COVID-19* Coronavirus Disease 2019, *OR* odds ratio

Variables included in the multivariate linear regression model (a): age, years of experience, work region (Others include North of Lebanon, South of Lebanon and Bekaa), occupation, comorbidities, dentist’s role about teaching others about COVID-19, afraid of getting infected with COVID-19, afraid about family members to get infected with COVID-19 due to occupational exposure

Variables included in the multivariate logistic regression model (b): gender, work region (Others include North of Lebanon, South of Lebanon and Bekaa), specialty, living with family members, dentist’s role about teaching others about COVID-19, afraid of getting infected with COVID-19, afraid about family members to get infected with COVID-19 due to occupational exposure, knowledge index

### Factors associated with high level of prevention measures towards COVID-19

The multivariate logistic regression model showed that dental specialists excluding surgeons were less likely to have high prevention measures level comparing to general dental practitioners. Besides, participants working in Mount Lebanon were less likely to have high prevention measures level comparing to those working in Beirut. However, dentists living with family members (OR = 2.3) and those who considered dentist’s role important in teaching others about COVID-19 (OR = 2.76) were more likely to have high prevention measures level. Lastly, having a higher knowledge index was significantly associated with a higher likelihood of having high prevention measures level (OR = 1.15) (Table 5b).

## Discussion

In this study, Lebanese dentists achieved an average knowledge index of 65% and an average prevention measures index of 71%. Scoring high level of prevention measures was associated with higher knowledge index, considering dentists role significant in teaching about COVID-19, living with family members, and being afraid about family members to get infected with the virus. Dental specialists were less likely to achieve high prevention measures index compared to general dental practitioners.

Our multivariate linear model showed that being younger was associated with better knowledge. Findings from other studies showed no association with age [14–16]. First, this difference in results may be partly related to the difference in the questions used to assess dentists’ knowledge. Second, we think that Lebanese young dentists were more knowledgeable than older ones because given the lack of national guidelines for dental healthcare in Lebanon before the start of the study, younger dentists who are more familiar with online learning have consulted the international guidelines that have been published on the international health institutions websites earlier since the start of the pandemic. Our study also showed that dentists working in Mount Lebanon region had higher knowledge score than those working in Beirut. This might be related to the significantly younger age of the dentists working in Mount Lebanon comparing to those working in Beirut (mean = 43 and 48 years old respectively). Lastly, dentists who considered their role significant in teaching others about COVID-19 achieved higher level of knowledge. Although dentists and physicians deliver different type of medical services, their trainings and education share similarities. Therefore, with their knowledge, dental professionals can contribute alongside other healthcare professionals to the fight against COVID-19 pandemic that poses a real challenge to worldwide healthcare systems. For instance, dental professionals should help in patients screening, should develop online platform to spread awareness about COVID-19 and educate the public about oral hygiene especially during lockdown periods.

Our study showed that dentists adopt high level of prevention measures in dental clinics during the pandemic including hand hygiene, surface disinfection, use of PPE and taking patient’s temperature as part of the triage process. This practice was similar to previous studies [17–25] and conform with the international [7, 8] and LDA guidelines [10]. However Lebanese dentists’ prevention practices were suboptimal mainly regarding rubber dam use and patients triage including questions about patient’s recent travel and patient’s contact with a person with suspected or known COVID-19 infection. Patient triage is crucial in order to identify the patients with suspected or confirmed COVID-19 infection and to schedule the dental care according to the emergency of the patients dental problem and to assess the need in terms of protection equipment and disinfection [10, 26].

Our multivariate logistic regression model showed that better knowledge was associated with having high prevention measures index. To our knowledge, only two previous studies explored the factors associated with prevention practices of dental professionals and reported similar association with knowledge [19, 23]. Unlike the previous study in Lebanon [23], the present study showed that specialists dentists were less likely to achieve high prevention measures index compared to general dental practitioners. We should note that the previous study was based on a snowball sampling technique and might have suffered from lack of representativeness for dental professionals in Lebanon. Working in Mount Lebanon was also associated with lower prevention measures index compared to working in Beirut. This might be explained in part by the younger age of dentists in Mount Lebanon, who may, because of their high knowledge, be more confident when taking more risk regarding the precautionary measures while delivering dental care to their patients. On the other hand, high level of prevention measures practice was associated with considering that dentist’s role is significant in teaching about COVID-19, living with family members, and being afraid about family members to get infected with the virus. Like other healthcare professionals, dentists worry that they might carry the virus home to their families. And therefore, they tend to take the essential prevention measures and to adhere to infection control protocol to avoid the transmission of the virus to their families.

On one hand around half of Lebanese dentists in the present study provided emergency treatment for their patients. The percentage of dentists providing emergency interventions was low in India [17, 25] and high in Turkey [15, 21]. This discrepancy might be explained by the availability or the lack of local guidelines and recommendations for dentists regarding the delivery of emergency therapies. In addition, Lebanese dentists opened their clinics on average 1 day per week reflecting a compliance with the total lockdown imposed by the Lebanese authorities. On the other hand, around half of the participants accepted to treat patients with flu like symptoms and patients with known or suspected COVID-19 infection. Most of dentists from previous studies mainly conducted in the early period of the COVID-19 outbreak prefer not to treat patients with COVID-19 [18, 22, 27, 28]. This was expected as many guidelines stated that only emergency interventions to be performed, with rescheduling of the elective treatment procedures [7, 9]. These findings may be attributed to the high risk of contamination of different areas of dentist’s face during dental practices [29], the inefficacy of standard protective measures in daily clinical work to prevent the spread of COVID-19 [30], the time during which studies were conducted (early during the outbreak), if dental management and infection control guidelines for COVID-19 from dental associations were available and lastly the classification of dentistry as a very high-risk occupation for COVID-19 infection according to the Occupational Safety and Health Act Guidance [31].

More than half of the dentists considered COVID-19 as a dangerous infection and were afraid of getting infected with COVID-19 due to occupational exposure and most of them were afraid about transmitting the virus to their family members. On one hand, there was no effective treatment or vaccine against COVID-19 at the time of the study. On the other hand, the average age for dentists who considered COVID-19 very dangerous was 48 years old in our study compared to an average of 43 years old among those who considered COVID-19 as not dangerous or moderately dangerous. Thus, older dentists are scared from being infected because increased age was shown to be associated with high risk of severe disease and complications [32].

One strength of our study is that it is the first study conducted among dentists in Lebanon with a representative sample randomly selected from the list of dentists registered in the LDA. Besides, the survey collected an important number of variables to identify factors related to knowledge and prevention practices of dentists regarding COVID-19. This study was one of the few that explored the factors related to adherence to prevention measures in dental clinics.

One limitation of this study is the lack in the knowledge questionnaire of questions related to the type of masks used, the use of mouthwash before and/or during dental care delivery and the choice of disinfectant solutions. Other limitations included recall and information bias related to the use of an online self-administered questionnaire as survey instrument.

## Conclusions

As information about COVID-19 continues to evolve rapidly, further improvement and update would be beneficial to optimally manage patients in this challenging situation. The knowledge on infection control in dental clinics should be strengthened through online educational trainings if physical meetings are not possible due to recurrent lockdowns. National guidelines for dental care should be regularly updated and shared with dentists particularly older ones.

## Data Availability

The datasets generated and analyzed during the current study are not publicly available due to limitations of ethical approval involving the patient data and anonymity but are available from the corresponding author on reasonable request.

## Abbreviations

COVID-19: Coronavirus disease 2019
LDA: Lebanese Dental Association
PPE: Personal protective equipment
SARS-CoV-2: Severe acute respiratory syndrome coronavirus 2
SD: Standard deviation
